# No Adaptation of the Prion Strain in a Heterozygous Case of Variant Creutzfeldt-Jakob Disease 

**DOI:** 10.3201/eid2606.191116

**Published:** 2020-06

**Authors:** Aileen Boyle, Chris Plinston, Fraser Laing, Graeme Mackenzie, Robert G. Will, Jean C. Manson, Abigail B. Diack

**Affiliations:** The Roslin Institute, Easter Bush, Scotland, UK (A. Boyle, C. Plinston, F. Laing, A.B. Diack);; Western General Hospital, Edinburgh, Scotland, UK (G. Mackenzie);; University of Edinburgh, Edinburgh (R.G. Will, J.C. Manson)

**Keywords:** Bovine spongiform encephalopathy, prions and related diseases, strain, variant Creutzfeldt-Jakob disease, transmissible spongiform encephalopathy, zoonoses, vCJD

## Abstract

We investigated a clinical case of variant Creutzfeldt-Jakob Disease in a person heterozygous for methionine/valine at codon 129 of the prion protein gene and identified the same strain properties in variant Creutzfeldt-Jakob disease in methionine homozygous persons and in bovine spongiform encephalopathy. These results indicate no adaptation of the agent in a different genetic background.

In 2016, a definite case of clinical variant Creutzfeldt-Jakob disease (vCJD) in a person heterozygous for methionine/valine (MV) at codon 129 of the prion protein gene (*PRNP* 129MV) was reported in the United Kingdom (*1*). Given the relatively atypical clinical features in this case, we considered it important to ascertain the strain of prion agent to determine whether there had been strain adaption or whether the patient’s genetic background may have influenced the disease phenotype. We conducted a study to determine whether we could isolate the same prion strain from this case of vCJD in a 129MV individual as was identified in previous 129 methionine homozygous (129MM) genotype vCJD cases, consistent with the hypothesis of a causal link to bovine spongiform encephalopathy (BSE). 

The clinical features for this patient were consistent with a diagnosis of either vCJD or sporadic Creutzfeldt-Jakob disease (sCJD). Results from magnetic resonance imaging (MRI) of the patient’s brain were suggestive of sCJD on diffusion-weighted imaging (DWI) sequences, although the single coronal fluid-attenuated inversion recovery (FLAIR) sequence in this case was not diagnostic because of movement artifact. Results of cerebrospinal fluid (CSF) real-time quaking-induced conversion assay analysis and the direct detection assay for vCJD infection in the blood were negative. However, at autopsy, neuropathological examination revealed florid plaques, and biochemical analysis of prion protein (PrP) from the brain confirmed a type 2B profile, both characteristic of vCJD (*1*). Abnormal PrP was also detected in peripheral tissues. Recent studies in which researchers used protein misfolding cyclic amplification in CSF were positive in this case of vCJD, but not in sCJD cases, including those with a heterozygous genotype (*2*). 

## The Study 

We injected 18 RIII mice with 10% wt/vol frozen central nervous system tissue, 0.02 mL intracerebrally and 0.1 mL intraperitoneally, from a 129MV patient with a clinical case of vCJD (*1*). The vCJD tissue samples were provided by the NHS National Prion Clinic, University College London (UCL) Hospitals (London, UK), and MRC Prion Unit at UCL and sourced through the MRC Edinburgh Brain Bank (Edinburgh, Scotland, UK). The Brain Bank has full ethics approval and consent for the use of tissue in research (East of Scotland Research Ethics Service, Ref 16/ES/0084) and works within the framework of the Human Tissue (Scotland) Act 2006. We conducted inoculation, clinical scoring, and neuropathological and biochemical analysis of the mice as previously described (*3*–*5*). Animal studies were conducted according to the regulations of the UK Home Office Animals (Scientific Procedures) Act 1986. 

The isolate from the brain of the 129MV patient transmitted successfully; clinical and neuropathological signs associated with prion disease appeared in the mice. We compared the mean incubation period, neuropathological signs, and biochemical analysis with archived records of UK 129MM vCJD central nervous system transmissions and UK BSE transmissions. Methods used for inoculation, clinical scoring, and neuropathological and biochemical analysis of the mice were described in previous publications (*3*–*5*). 

Clinical signs with individual incubation periods ranging from 300 to 392 days postinfection (dpi) were apparent in the mice. The major clinical signs were a loss of body weight and body condition with eye winking and gait abnormalities. Toward the end of the clinical phase, a wet genital area could also be observed. Pathologically confirmed disease developed in 14 of 16 mice (mean incubation period + SEM 341 + 6 dpi). This finding is within the range of previous transmission studies for UK vCJD in this mouse line (mean incubation 306–387 dpi) and similar to those for BSE (mean incubation 316–335 dpi). 

**Figure 1:**
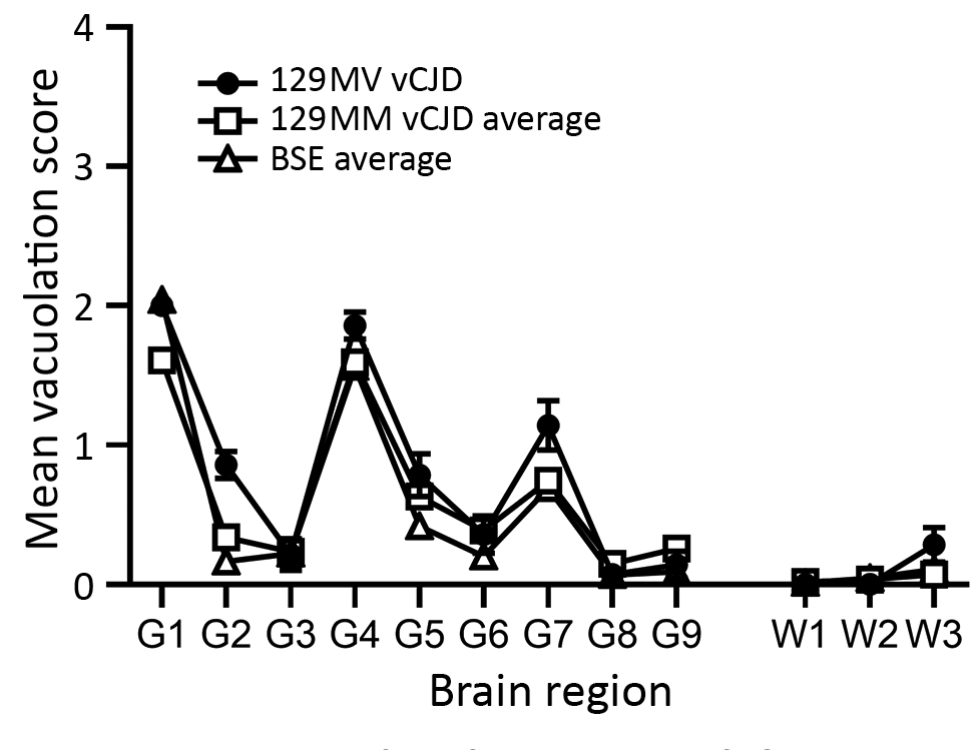
Vacuolation profiles of a clinical case of vCJD in a prion protein gene codon 129MV individual, pooled data from UK 129MM cases (n = 7) and pooled data from UK bovine spongiform encephalopathy cases (n = 8) show similarities in vacuolar pathology intensity and distribution in wild-type mouse brains. Data show mean ± SEM of clinical and pathological positive mice (n≥6 per group). G1–G9, gray matter scoring regions: G1, medulla; G2, cerebellum; G3, superior colliculus; G4, hypothalamus; G5, thalamus; G6, hippocampus; G7, septum; G8, retrosplenial and adjacent motor cortex; G9, cingulate and adjacent motor cortex. W1–W3, white matter scoring regions: W1, cerebellar white matter; W2, mesencephalic tegmentum; W3, cerebral peduncle. MM, methionine homozygous; MV, methionine/valine; vCJD, variant Creutzfeldt-Jakob disease.

**Figure 2:**
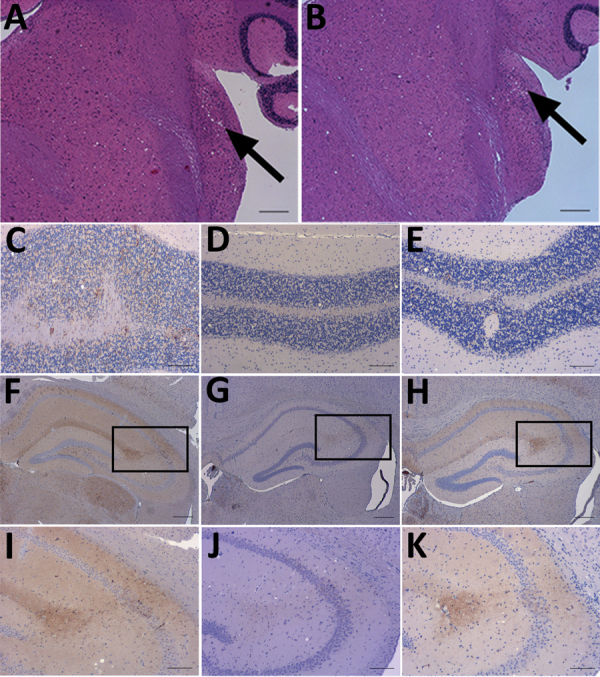
Neuropathology of RIII mice inoculated with material from a clinical case of vCJD in a prion protein gene codon 129MV individual, a typical 129MM case of vCJD, and BSE. A, B) Haemotoxylin and Eosin staining of transmissible spongiform encephalopathy vacuolation in the cochlear nucleus of mice inoculated with material from a clinical 129MV case (arrows). C–E) Abnormal PrP deposition in the cerebellum of mice inoculated with C) clinical 129MV case, D) typical 129MM case, and E) BSE. F–H) Abnormal PrP deposition in the hippocampus of mice inoculated with samples of F) clinical 129MV case, G) typical 129MM case, and H) BSE. I–K) Abnormal PrP deposition in the CA2 region of the hippocampus; I) inset from panel F; J) inset from panel G; K) inset from panel H. Monoclonal antibody: 6H4. Scale bars: A–B, F–H: 200 µm. C–E, I–K: 100 µm. BSE, bovine spongiform encephalopathy; MM, methionine homozygous; MV, methionine/valine; PrP, prion protein; vCJD, variant Creutzfeldt-Jakob disease.

We also generated a transmissible spongiform encephalopathy (TSE) vacuolation profile from clinically affected RIII mice and compared it with profiles from UK vCJD and BSE transmissions (Figure 1). We observed a mild-to-moderate gray matter vacuolation in the medulla, hypothalamus, and septum and moderate vacuolation in the cochlear nucleus and dorsal raphe (Figure 1; Figure 2, panels A, B). 

We conducted an immunohistochemical analysis, which showed abnormal PrP deposition throughout the brain of both a granular and punctate nature (Figure 2, panels C–K). There was heavy staining in the brainstem, particularly the superior vestibular and cochlear nuclei, and lower midbrain, where the substantia nigra was often targeted. Most of the thalamic nuclei exhibited staining, but staining was more intense in the habenular, hypothalamus, and the CA2 region of the hippocampus (Figure 2, panels F–K). Punctate staining was also apparent in the mid-layer of the cortex throughout the brain. This pattern of staining was very similar to that observed in a vCJD and BSE transmission in the United Kingdom, with additional observations of granular deposition in the cerebellar cortex and small plaques occurring in the corpus callosum in 2 of the samples. 

Biochemical analysis of the MV isolate confirmed the presence of protease-resistant PrP (PrP^res^). We identified a similar type 2B–like pattern and glycosylation profile in the RIII mice. This profile is characterized by a predominance of the diglycosylated form of PrP^res^ at ≈30 kDa, a monoglycosylated form at ≈27kDa, and an unglycosylated band at ≈19kDa (Appendix Figure). The biochemical profile appears identical between the RIII-MV, RIII-MM, and RIII-BSE isolates tested, although the RIII-BSE isolate appeared to have less PrP^res^. 

## Conclusions

This transmission study in RIII mice provides evidence that the prion strain isolated from this confirmed case of vCJD in a 129MV person is the same as that identified in typical 129MM vCJD and BSE cases. Further characterization in a range of mouse models is ongoing. However, transmission to RIII mice in previous studies has led to definitive identification of several strains, vCJD and BSE in particular (*6*,*7*). 

*PRNP* codon 129 genotype has been shown to be a major factor influencing disease characteristics of Creutzfeldt-Jakob disease (*8*), but it has not been established if the same is true of vCJD, because previous vCJD infections in 129MV persons exposed to contaminated blood products have been asymptomatic (*9*,*10*). Earlier studies using gene-targeted mice inoculated with vCJD predicted that codon 129 genotype would determine disease susceptibility and incubation periods (*11*), whereas other transgenic mouse studies demonstrated that BSE could transmit with a different phenotype in mice expressing 129MV than that observed in mice expressing 129MM (*12*). 

The clinical diagnosis in the MV case we report was uncertain while the patient was alive, and it was only at autopsy that neuropathology and biochemistry confirmed vCJD. The neurologic features alone cannot be used to discriminate between sCJD and vCJD, and the MRI findings on DWI imaging favored a diagnosis of sCJD. However, the high sensitivity and specificity of MRI for vCJD were determined by analyzing FLAIR images primarily (*13*) and recent review suggests that DWI imaging may be less specific than FLAIR imaging in vCJD. It is possible that the phenotype of vCJD in this case may have been altered by the heterozygous *PRNP* background and investigations including CSF protein misfolding cyclic amplification (*2*), tonsil biopsy, and perhaps FLAIR MRI may contribute to accurate diagnosis of future heterozygous cases. 

The identification of vCJD in a 129MV person may indicate the start of a second wave of vCJD in association with the 129MV genotype which is present in around 45% of the UK population (*14*), although no further cases have been reported since 2016. This case highlights the need to continue surveillance to identify new cases of vCJD and the need for autopsy and strain typing in persons with prion diseases. Changes in clinical disease phenotype could mask the true diagnosis and may be indicative of potential changes in prion disease strains and infectious properties. Strain identification and assessing the infectious properties of prion diseases are essential components in the management of these diseases and have important implications for public health and in determining the prevalence of BSE-related prion disease in humans. 

Appendix Additional information for study showing no adaption of the prion strain in a heterozygous case of variant Creutzfeldt-Jakob disease.

## References

[1] Mok T, Jaunmuktane Z, Joiner S, Campbell T, Morgan C, Wakerley B, et al. Variant Creutzfeldt-Jakob disease in a patient with heterozygosity at PRNP codon 129. N Engl J Med. 2017;376:292–4. 10.1056/NEJMc1610003 28099827

[2] Bougard D, Bélondrade M, Mayran C, Bruyère-Ostells L, Lehmann S, Fournier-Wirth C, et al. Diagnosis of methionine/valine variant Creutzfeldt-Jakob disease by protein misfolding cyclic amplification. Emerg Infect Dis. 2018;24:1364–6. 10.3201/eid2407.172105 29912702 PMC6038758

[3] Wiseman FK, Cancellotti E, Piccardo P, Iremonger K, Boyle A, Brown D, et al. The glycosylation status of PrPC is a key factor in determining transmissible spongiform encephalopathy transmission between species. J Virol. 2015;89:4738–47. 10.1128/JVI.02296-14 25673720 PMC4403468

[4] Dickinson AG, Meikle VMH, Fraser H. Identification of a gene which controls the incubation period of some strains of scrapie agent in mice. J Comp Pathol. 1968;78:293–9. 10.1016/0021-9975(68)90005-4 4970191

[5] Fraser H, Dickinson AG. The sequential development of the brain lesion of scrapie in three strains of mice. J Comp Pathol. 1968;78:301–11. 10.1016/0021-9975(68)90006-6 4970192

[6] Bruce ME, Will RG, Ironside JW, McConnell I, Drummond D, Suttie A, et al. Transmissions to mice indicate that ‘new variant’ CJD is caused by the BSE agent. Nature. 1997;389:498–501. 10.1038/39057 9333239

[7] Bruce ME. TSE strain variation. Br Med Bull. 2003;66:99–108. 10.1093/bmb/66.1.99 14522852

[8] Parchi P, Strammiello R, Notari S, Giese A, Langeveld JP, Ladogana A, et al. Incidence and spectrum of sporadic Creutzfeldt-Jakob disease variants with mixed phenotype and co-occurrence of PrP^Sc^ types: an updated classification. Acta Neuropathol. 2009;118:659–71. 10.1007/s00401-009-0585-1 19718500 PMC2773124

[9] Peden A, McCardle L, Head MW, Love S, Ward HJ, Cousens SN, et al. Variant CJD infection in the spleen of a neurologically asymptomatic UK adult patient with haemophilia. Haemophilia. 2010;16:296–304. 10.1111/j.1365-2516.2009.02181.x 20070383

[10] Peden AH, Head MW, Ritchie DL, Bell JE, Ironside JW. Preclinical vCJD after blood transfusion in a PRNP codon 129 heterozygous patient. Lancet. 2004;364:527–9. 10.1016/S0140-6736(04)16811-6 15302196

[11] Bishop MT, Hart P, Aitchison L, Baybutt HN, Plinston C, Thomson V, et al. Predicting susceptibility and incubation time of human-to-human transmission of vCJD. Lancet Neurol. 2006;5:393–8. 10.1016/S1474-4422(06)70413-6 16632309

[12] Asante EA, Linehan JM, Gowland I, Joiner S, Fox K, Cooper S, et al. Dissociation of pathological and molecular phenotype of variant Creutzfeldt-Jakob disease in transgenic human prion protein 129 heterozygous mice. Proc Natl Acad Sci U S A. 2006;103:10759–64. 10.1073/pnas.0604292103 16809423 PMC1502304

[13] Collie DA, Summers DM, Sellar RJ, Ironside JW, Cooper S, Zeidler M, et al. Diagnosing variant Creutzfeldt-Jakob disease with the pulvinar sign: MR imaging findings in 86 neuropathologically confirmed cases. AJNR Am J Neuroradiol. 2003;24:1560–9. 13679271 PMC7973975

[14] Bishop MT, Pennington C, Heath CA, Will RG, Knight RS. PRNP variation in UK sporadic and variant Creutzfeldt Jakob disease highlights genetic risk factors and a novel non-synonymous polymorphism. BMC Med Genet. 2009;10:146. 10.1186/1471-2350-10-146 20035629 PMC2806268

