# Supplementary material for: No Adaptation of the Prion Strain in a Heterozygous Case of Variant Creutzfeldt-Jakob Disease
Source: Emerg Infect Dis. 2020 Jun;26(6):1300–3. doi: 10.3201/eid2606.191116 (PMC7258451; doi:10.3201/eid2606.191116)
Supplement: Appendix — Additional information for study showing no adaption of the prion strain in a heterozygous case of variant Creutzfeldt-Jakob disease. [file 19-1116-Techapp-s1.pdf]

Article DOI: https://doi.org/10.3201/eid2606.191116

No Adaptation of the Prion Strain in a Heterozygous Case of Variant Creutzfeldt-Jakob Disease

Appendix

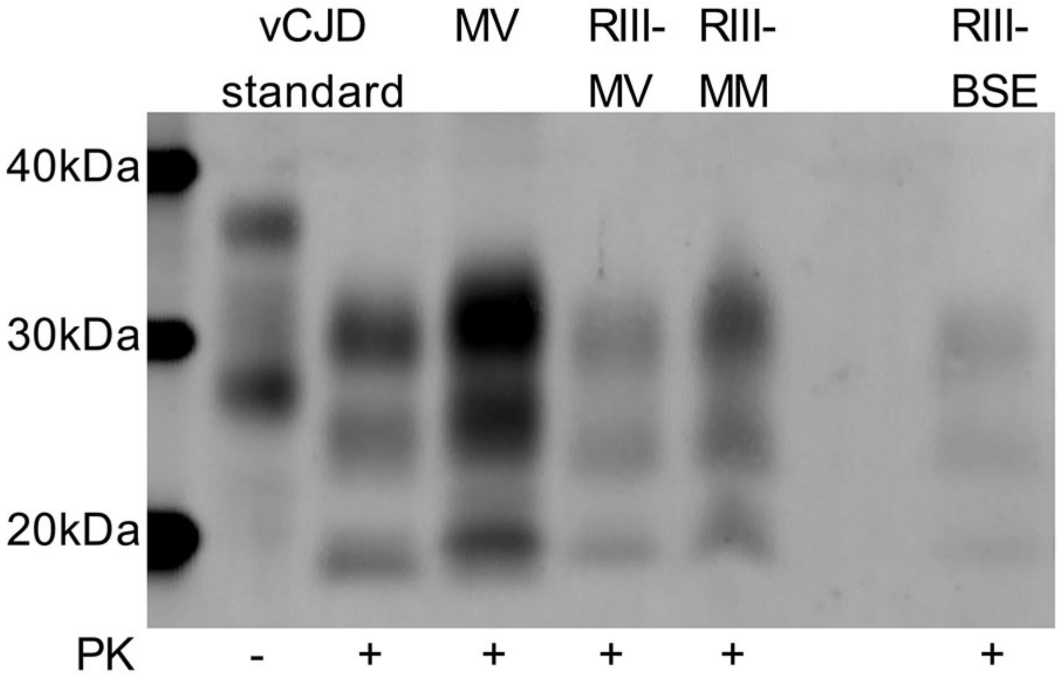

Appendix Figure. Western blot analyses of proteinase K resistant isoforms of PrP (PrP^{res}) show similar Type 2B like profiles in representative RIII mice inoculated with the 129MV case, a typical 129MM case and BSE. A typical 129MM vCJD standard and the 129MV isolate are shown for reference. Blots were probed with monoclonal antibody 6H4 (1:5000). Approximate molecular mass is shown in kDa.
